# N-Glycosylation of Human R-Spondin 1 Is Required for Efficient Secretion and Stability but Not for Its Heparin Binding Ability

**DOI:** 10.3390/ijms17060937

**Published:** 2016-06-14

**Authors:** Chiung-Fang Chang, Li-Sung Hsu, Chieh-Yu Weng, Chih-Kai Chen, Shu-Ying Wang, Yi-Hwa Chou, Yan-Yu Liu, Zi-Xiu Yuan, Wen-Ying Huang, Ho Lin, Yau-Hung Chen, Jen-Ning Tsai

**Affiliations:** 1 Department of Life Sciences, National Chung Hsing University, Taichung 40227, Taiwan; d099052009@mail.nchu.edu.tw (C.-F.C.); hlin@dragon.nchu.edu.tw (H.L.); 2 Institute of Biochemistry and Biotechnology, Chung Shan Medical University, Taichung 40201, Taiwan; lsh316@csmu.edu.tw; 3 Clinical Laboratory, Chung Shan Medical University Hospital, Taichung 40201, Taiwan; 4 Department of Medical Laboratory and Biotechnology, Chung Shan Medical University, Taichung 40201, Taiwan; elsie1009@hotmail.com (C.-Y.W.); okai1025@hotmail.com (C.-K.C.); lucywang0515@gmail.com (S.-Y.W.); a26001234@gmail.com (Y.-H.C.); xprfish1210@gmail.com (Y.-Y.L.); a12456910@gmail.com (Z.-X.Y.); f920719@yahoo.com.tw (W.-Y.H.); 5 Department of Chemistry, Tamkang University, Tamsui, New Taipei City 25137, Taiwan; yauhung@mail.tku.edu.tw

**Keywords:** R-spondin 1, N-glycosylation, secretion, stability, Wnt signaling

## Abstract

R-spondin 1 (Rspo1) plays an essential role in stem cell biology by potentiating Wnt signaling activity. Despite the fact that Rspo1 holds therapeutic potential for a number of diseases, its biogenesis is not fully elucidated. All Rspo proteins feature two amino-terminal furin-like repeats, which are responsible for Wnt signal potentiation, and a thrombospondin type 1 (TSR1) domain that can provide affinity towards heparan sulfate proteoglycans. Using chemical inhibitors, deglycosylase and site-directed mutagenesis, we found that human Rspo1 and Rspo3 are both N-glycosylated at N137, a site near the C-terminus of the furin repeat 2 domain, and Rspo2 is N-glycosylated at N160, a position near the N-terminus of TSR1 domain. Elimination of N-glycosylation at these sites affects their accumulation in media but have no effect on the ability towards heparin. Introduction of the N-glycosylation site to Rspo2 mutant at the position homologous to N137 in Rspo1 restored full glycosylation and rescued the accumulation defect of nonglycosylated Rspo2 mutant in media. Similar effect can be observed in the N137 Rspo1 or Rspo3 mutant engineered with Rspo2 N-glycosylation site. The results highlight the importance of N-glycosylation at these two positions in efficient folding and secretion of Rspo family. Finally, we further showed that human Rspo1 is subjected to endoplasmic reticulum (ER) quality control in N-glycan-dependent manner. While N-glycan of Rspo1 plays a role in its intracellular stability, it had little effect on secreted Rspo1. Our findings provide evidence for the critical role of N-glycosylation in the biogenesis of Rspo1.

## 1. Introduction

R-spondins (roof plate-specific spondins, Rspos) belong to a family of four secreted proteins capable of potentiating Wnt signaling activity. The link between Rspos and Wnt signaling was originally identified by the co-expression of Wnt and Rspos in the dorsal neural tube (roof plate) during embryonic development [1] and later characterized in *Xenopus*, where Rspo2 was identified as an activator of canonical Wnt signaling [2]. All members of the Rspo family contain an N-terminal signal peptide (SP), two furin-repeat domains that are rich in cysteine residues (FR1 and FR2; also called cysteine-rich domain, CRD), one thrombospondin type 1 domain (TSR1), and a C-terminal region enriched with positively charged amino acids (CT). The CRD is often found in secretory proteins and transmembrane receptors, and recent studies have shown that this domain is necessary and sufficient to enhance canonical Wnt signaling activity [2,3,4].

The roles of Rspos during development have been elucidated by studying inherited genetic diseases caused by mutations in this gene family or through the use of knockout or knockdown animal models. Homozygous mutations of human *Rspo1* result in hermaphroditism, palmoplantar hyperkeratosis and an increased risk of squamous cell skin carcinoma [5]. Mutations in the human *Rspo4* gene lead to congenital anonychia, an anomaly manifested by the absence or hypoplasia of nails [6,7]. *Rspo2*-null mice displayed phenotypes including limb and craniofacial anomalies, and lung hypoplasia [8,9], whereas knockdown of *Rspo2* in *Xenopus* embryos resulted in defective myogenesis [2]. *Rspo3*-deficient mice are embryonic lethal due to vascular defects in the placenta [10]. Further analysis by gain- and loss-of-function approaches in mouse and *Xenopus* indicated that *Rspo3* is essential for angioblast specification and vascular development [11]. In addition to their roles during embryonic development, Rspos also play diverse roles in physiological processes in adult vertebrates. For example, Rspo1 was shown to possess potent mitogenic effects on Wnt-dependent adult intestinal stem cells both *in vivo* [12] and *in vitro* [13,14]. As a consequence, Rspo1 can function as a stem cell growth factor and thus holds therapeutic potential for the treatment of gastrointestinal diseases.

The molecular mechanism by which Rspos potentiate Wnt signaling was controversial until the identification of type 2 leucine-rich repeat-containing G-protein-coupled receptors (Lgr4, 5 and 6) as the high-affinity receptors for Rspos [15,16,17]. Recent studies further indicated that the transmembrane RING finger ubiquitin ligase Zinc and RING finger 3 (Znrf3) and related Ring finger 43 (Rnf43) are associated with Wnt receptors and Rspo ligands, which established a novel mechanism of Rspo action [18,19]. In the absence of Rspo ligands, Rnf43 and Znrf3 promote turnover of Frizzled and Lrp5/6 receptors by selective ubiquitination, thereby reducing Wnt signals. Rspo ligands exert their function by interacting with the extracellular domains of Lgr4/5/6 and Znrf3/Rnf43, which induces the clearance of Znrf3/Rnf43 from the membrane and thereby stabilizes the receptors to potentiate Wnt signaling. Crystallographic studies using the CRD domains of Rspo1 and Rspo2 revealed their basic architectures and their interaction with receptors [3,20,21,22,23,24]. The two furin repeats in the CRD adopt a ladder-like structure of β-hairpins, and each furin domain is comprised of three β-hairpins connected by four disulphide bridges. Overall, the CRD domain is characterized by a head module and a rod module; the head module interacts with Znrf3/Rnf43, while the rod module binds Lgr4/5/6. Several lines of evidence have suggested that TSR1 and the C-terminal regions could facilitate interactions with heparan sulfate proteoglycans (HSPGs) located on the cell surface and in the extracellular matrix. Indeed, the deletion of the TSR and CT domains from mouse Rspo3 resulted in significantly lower affinity for heparin [4]. Positively charged surface amino acids in the TSR1 and CT domains may contribute to heparin binding. Recently, the TSR1 domain of Rspo3 was shown to bind syndecan 4, confirming an interaction between the Rspo proteins and HSPGs [25]. In addition, calorimetric measurements indicated that a Rspo1 fragment containing both the CRD and TSR1 domains displays two-fold higher affinity toward Rnf43 when compared with the Rspo1 CRD domain alone [23]. This finding suggests that the TSR1 domain also contributes to the stability of its receptor complex.

Protein N-glycosylation, a common type of co- or post-translational modification, is essential for many protein functions, such as protein folding and quality control in the endoplasmic reticulum (ER), secretion, and several biological recognition events [26]. Most secretory proteins undergo N-linked glycosylation during their ER transit. The basic organization of the modification pathway is conserved across a wide range of species and involves the attachment of a dolichol lipid-linked oligosaccharide precursor to the Asn residue of the Asn-Xaa-Ser/Thr motif (where Xaa can be any amino acid except Pro; also known as a sequon) in the polypeptide chain entering the ER lumen [27,28,29]. Eukaryotic organisms utilize the oligosaccharyltransferase (OST) on the luminal face of the ER membrane to catalyse this glycan transfer reaction [30]. Next, the core glycan undergoes further modifications by trimming two Glc residues using glycosidases in the ER. The resulting glycan structure provides ligands for lectin chaperones and contributes to quality control surveillance in the ER [31]. After leaving the ER, the glycoproteins move through the Golgi apparatus, where their glycans undergo further processing [32]. In brief, N-glycans on nascent glycoproteins play an essential role in protein secretion, as they affect protein folding, provide ligands for lectin chaperones, contribute to quality control surveillance in the ER and mediate transit and selective protein targeting throughout the secretory pathway [26,33].

Despite the fact that Rspo1 has been implicated as a potential therapy in a number of pathological processes, its biogenesis is yet to be elucidated. Based on the amino acid sequence, human Rspo1 contains one potential N-glycosylation consensus sequence (sequon) at position Asn137 (N137), located at the C-terminus of the FR2. This sequon is highly conserved in most Rspo family members, including Rspo1 and Rspo3. On the other hand, another sequon at position Asn160 (N160), located at the N-terminus of TSR1 domain, is present in all Rspo2 members. In the present study, we found that Rspo1 and 3 are N-glycosylated at N137 sites. Abrogation of N-glycosylation at this site affects their efficient folding and accumulation in media but has no impact on their binding ability on heparin. Rspo2 is N-glycosylated at N160, a sequon located in the TSR1 domain. Introduction of the N-glycosylation site to Rspo2 mutant at the position homologous to N137 in Rspo1 restored full glycosylation and rescued the accumulation defect of nonglycosylated Rspo2 mutant in media, and vice versa. Finally, we further showed that human Rspo1 is subjected to ER quality control in N-glycan-dependent manner. While N-glycan of Rspo1 plays a role in its intracellular stability, it had little effect on stability of secreted Rspo1.

## 2. Results

### 2.1. Human Rspo1 Is N-Glycosylated at Asn137 in Vivo

**Figure 1:**
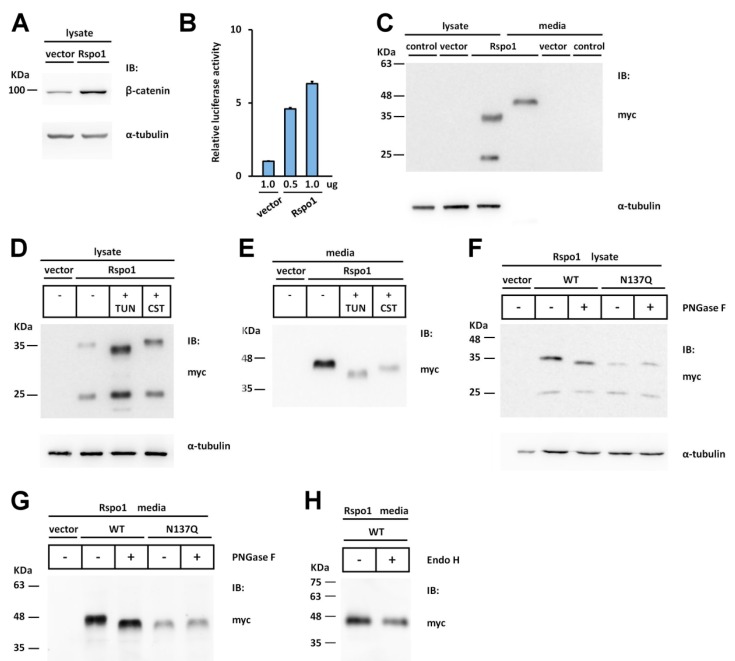
Human Rspo1 is N-glycosylated at Asn137. (**A**,**C**) Cell lysates or conditioned media from control vector or myc-tagged human Rspo1-transfected HEK293T cells were analyzed for endogenous soluble β-catenin (**A**) or the expression of myc-tagged human Rspo1 (**C**) by Western blot. (**C**) “Control” and “vector” indicated un-transfected control and empty vector-transfected control, respectively; (**B**) SuperTopFlash plasmids were co-transfected with either the indicated amounts of control vector or myc-tagged Rspo1-expressing plasmids. Renilla luciferase, driven by the thymidine kinase gene promoter, was included as a transfection control; (**D**,**E**) Human Rspo1 was transiently expressed in HEK293T cells in the presence of tunicamycin (TUN) or castanospermine (CST). The cell lysates (**D**, 15 μg per lane) and conditioned media (**E**, 23 μL per lane) were analyzed by Western blot; (**F**,**G**) The cell lysates (**F**) and conditioned media (**G**) of wild type (WT) human Rspo1 or N137Q mutant-transfected HEK293T cells were treated with (+) or without (−) PNGase F; (**H**) The conditioned media from WT Rspo1-transfected cells were treated with (+) or without (−) Endo H. α-tubulin was used as a loading control for the cell lysates in each blot.

Human *Rspo1-4* transcripts were previously detected in HEK293T cells, although *Rspo3* showed lower expression [2]. We therefore sought to study the expression pattern of human Rspo1 by expressing myc-tagged human Rspo1 in HEK293T cells. Expression of Rspo1 induced cytosolic β-catenin accumulation (Figure 1A), a hallmark of the activated Wnt/β-catenin pathway [34]. In addition, co-expression of Rspo1 with SuperTopFlash (STF), a reporter plasmid carrying tcf1-binding sites in the upstream regulatory regions of luciferase [35], in HEK293T cells also induced tcf1-dependent reporter activity (Figure 1B). This result suggested that myc-tagged human Rspo1 is biologically active in enhancing Wnt/β-catenin activity in HEK293T cells.

To detect the expression pattern of Rspo1 in HEK293T cells, cell lysates and conditioned media were subjected to Western blot analysis using an anti-myc antibody. Two immunoreactive bands of approximately 24 (the faster migrated band) and 36 kDa (the slower migrated band) and one 45 kDa band were observed in the cell lysates and conditioned media of transfected cells, respectively (Figure 1C). The predicted molecular weight of myc-tagged human Rspo1 is approximately 32 kDa without any post-translational modification. Because the expressed proteins (the slower migrated band in cell lysate and the band in conditioned media) showed larger molecular masses than expected, we speculated that Rspo1 might undergo post-translational modification in cells. As N-glycosylation is one of the most common post-translational modifications of proteins, we hypothesized that the difference between the predicted and apparent molecular weight of Rspo1 was caused by glycosylation. To test this hypothesis, we treated the transfected cells with an N-glycosylation inhibitor, tunicamycin, or a glucosidase inhibitor, castanospermine. Tunicamycin is an antibiotic that blocks the reaction of UDP-GlcNAc and dolicholphosphate, thereby inhibiting the synthesis of all N-linked glycoproteins [36]. Castanospermine is an indolizine alkaloid that suppresses the activity of α-glycosidase, leading to failure of glucose trimming and thereby preventing glycan-dependent association with the ER lectin chaperones, such as calnexin and calreticulin [37,38]. With the addition of tunicamycin, the slower migrated band in the cell lysate and the major band in conditioned media both displayed a reduced molecular mass (Figure 1D,E), suggesting that Rspo1 is an N-linked glycoprotein. In addition, the amount of secreted Rspo1 was reduced (Figure 1E). Following treatment with castanospermine, the amount of secreted Rspo1 into the conditioned media was also substantially affected in transfected cells (Figure 1E), highlighting the importance of the interaction with calnexin and calreticulin during Rspo1 processing in cells. To confirm the presence of N-glycosylation in Rspo1, we further treated the expressed Rspo1 with peptide N-glycosidase F (PNGase F), an amidase that cleaves between the innermost GlcNAc and Asn residues of oligosaccharides from N-linked glycoproteins to release Asn-linked glycans [39]. Treatment of the cell lysates and the conditioned media with PNGase F markedly reduced the molecular mass of the slower migrated band in the cell lysate and the major band in the conditioned media (Figure 1F,G), confirming the results described above. However, secreted Rspo1 was resistant to the digestion of Endo H (Figure 1H), which cleave N-linked glycoproteins with high mannose and hybrid glycans. This suggested that secreted Rspo1 is composed of complex oligosaccharides. Together, these results indicate that Rspo1 is N-glycosylated and that this modification is required for Rspo1 secretion in HEK293T cells.

While treatment of tunicamycin or PNGase F reduced the molecular mass of the slower migrated band in the expressed cell lysate, the treatment had no effect on the mobility of the faster migrated band, suggesting that this band might represent a form of Rspo1 without N-glycosylation. We hypothesized that the faster migrating band might be resulted from translation initiated at an internal methionine residue (Met 91). This would result in a 22.3 kDa fragment of Rspo1 (plus the epitope-tag). Since this C-terminal fragment will lack the signal sequence, it will remain in the cytosol.

**Figure 2:**
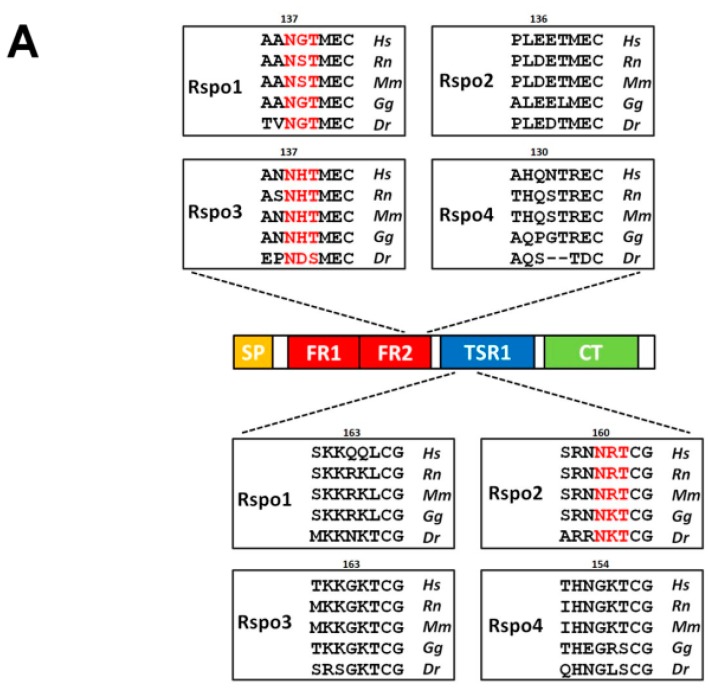

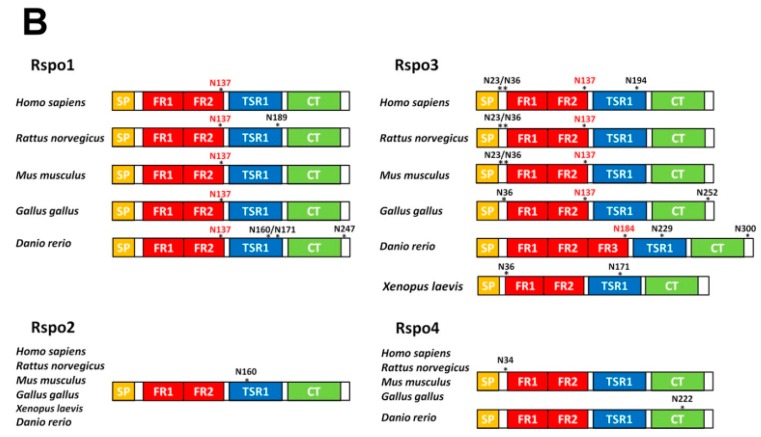
Location of the sequons in human Rspo family. (**A**) The location of conservative sequons (N137) among the vertebrate Rspo1 and Rspo3 family members and the unique sequon (N160) in Rspo2 members. All Rspo proteins contain an N-terminal signal peptide (SP), two furin-repeat domains (FR1 and FR2; also collectively known as the cysteine-rich domain, CRD), one thrombospondin type 1 domain (TSR1), and a C-terminal region enriched with positively charged amino acids (CT). One exception is that teleost Rspo3 members contain one additional furin repeat (FR3) (shown in **B**). Amino acid sequences located around the N137 (in Rspo1 and 3) and N160 (in Rspo2) are shown in boxes. Putative sequons are denoted in red. Hs: *Homo sapiens*; Rn: *Rattus norvegicus*; Mu: *Mus musculus*; Gg: *Gallus gallus*; Dr: *Danio rerio*; (**B**) Schematic illustration of the sequons in Rspo family. The potential sequons are marked by asterisks. The conserved N137 sites are shown in red.

Although the experiments using N-glycosylation inhibitors highlighted the importance of N-glycosylation in Rspo1 secretion, the precise site(s) of N-glycosylation within this protein are not yet determined. To localise the N-glycosylation site, we first searched for the potential N-glycosylation site (sequon) in Rspo1. The human Rspo1 protein consists of 263 amino acids with only one sequon at Asn 137, located in the C-terminal region in the FR2 domain (Figure 2A,B). This sequon is not only highly conserved in most of the Rspo1 members, but is also present in most Rspo3 family members (Figure 2A,B). However, it does not occur in this region in all Rspo2 and Rspo4 proteins (Figure 2A,B). To determine whether this putative N-glycosylation site is utilized *in vivo*, we eliminated this site in Rspo1 by substituting the Gln codon for the Asn codon, resulting in a N137Q mutant. When this N137Q mutant was expressed in HEK293T cells, the molecular mass of mutant N137Q in the cell lysates and the conditioned media showed a size reduction compared to wild-type (WT) Rspo1 (Figure 1F,G). This pattern of size reduction was similar to that observed in cell lysates and conditioned media from tunicamycin-treated cells transfected with WT Rspo1 (compare Figure 1F,G with Figure 1D,E), indicating that Rspo1 is N-glycosylated on Asn137. To further confirm this observation, we also treated the N137Q mutant with the deglycosylating enzyme PNGase F. In both the cell lysates and the conditioned media, PNGase F treatment had no effect on the molecular weight of the N137Q mutant (Figure 1F,G). Taken together, these results indicate that Rspo1 is N-glycosylated at Asn137.

While a difference of approximately 13 kDa exists between mature Rspo1 and unmodified Rspo1, the addition of N-glycans at N137 only caused an incremental increase in the molecular mass of approximately 2–3 kDa in secreted human Rspo1. This result suggests that other form(s) of post-translational modification exist in mature Rspo1.

### 2.2. Rspo1 N-Glycosylation at Asn137 Affects Its Secretion

**Figure 3:**
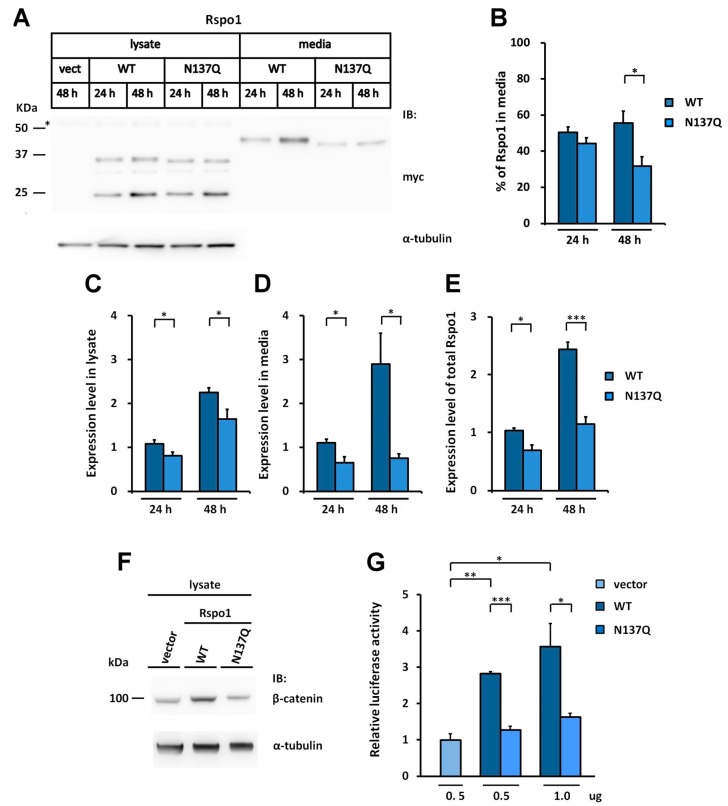
N-glycosylation of human Rspo1 enables efficient secretion from cells. (**A**) WT Rspo1 or N137Q mutant was transfected into HEK293T cells. After 24 or 48 h, the cell lysates and conditioned media were analyzed for myc-tagged Rspo1. Asterisk indicates non-specific bands; (**B**–**E**) Quantification of relative expression levels for human WT Rspo1 *versus* N137Q mutants in cell lysates, media and total. Immunoblots were quantified densitometrically and relative expression level of Rspo1 in media or lysate was calculated. Percentage of Rspo1 in media was defined as the Rspo1 in the media as a percentage of the total Rspo1 (lysates plus media). Error bars represent the mean ± SD; (**F**) WT Rspo1 or N137Q mutant was transfected into HEK293T cells. After 48 h, the cell lysates were analyzed for soluble β-catenin; (**G**) SuperTopFlash plasmids were co-transfected with the indicated amounts of control vector, WT or N137Q Rspo1-expressing plasmids. Renilla luciferase was included as a transfection control. Error bars represent the mean ± SD. All experiments were performed at least two times, in triplicate. The Student’s *t*-test was used to evaluate statistical significance (* *p* < 0.05; ** *p* < 0.01; *** *p* < 0.001). α-tubulin was used as a loading control for the cell lysates.

We noticed that the amount of secreted N137Q was lower than that of WT Rspo1 in the conditioned media of transfected HEK293T cells (Figure 1G). This observation is consistent with previous observations, showing that interference with the N-glycosylation process by tunicamycin or castanospermine led to secretory defects for human Rspo1 (Figure 1E). To investigate the best time point for comparison of secretion level, we performed a time course analysis of expression level for WT Rspo1 *versus* N137Q mutant at 24 and 48 h post-transfection. We quantitated the relative levels of Rspo1 in cell lysates, media and total (cell lysates plus media) (Figure 3A–E). At 24-h post-transfection, though the percentage of Rspo1 in media was similar in WT and N137Q Rspo1 (WT, 50%; N137Q, 44%), the relative expression levels in lysates, media and total of N137Q Rspo1 were slightly lower than that of WT (Figure 3A–E). However, after a longer incubation period, the N137Q mutant displayed a significant reduced level in media and the percentage of Rspo1 in media at 48 h post-transfection (% in media: WT, 56%; N137Q, 32%) (Figure 3A,B). Therefore the following experiments were all carried out at 48 h post-transfection, unless otherwise specified. To compare the amount of secreted WT Rspo1 and its mutant more clearly, total expression levels and the percentage of Rspo1 in media was presented in the following experiments.

Because secreted Rspo1 can act through an autocrine pathway to enhance Wnt/β-catenin activity [4], a reduced amount of secreted Rspo1 would lead to lower or no enhanced Wnt/β-catenin activity in transfected cells. Therefore, we also determined the activity of Rspo for stabilizing soluble β-catenin in transfected cells. In accordance with the secretion results, the level of soluble β-catenin in N137Q mutant-transfected cells was lower than in WT Rspo1-transfected cells (Figure 3F). In addition, when co-transfected with a STF reporter plasmid, WT Rspo1 was more active than N137Q mutant (Figure 3G). These results corroborated the above finding that N137Q Rspo1 is a secretory mutant. Taken together, these results indicate that N-glycosylation at N137 enables efficient secretion of Rspo1.

### 2.3. N-Glycosylation of Human Rspo3 Also Affects Its Secretion in Vivo

**Figure 4:**
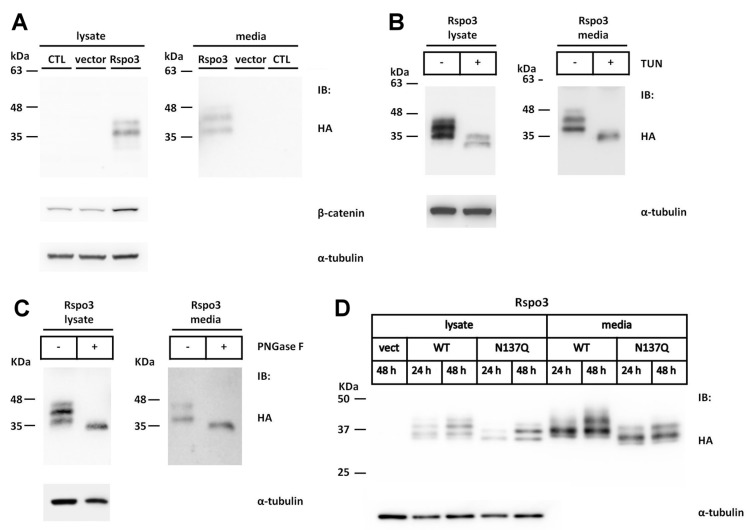

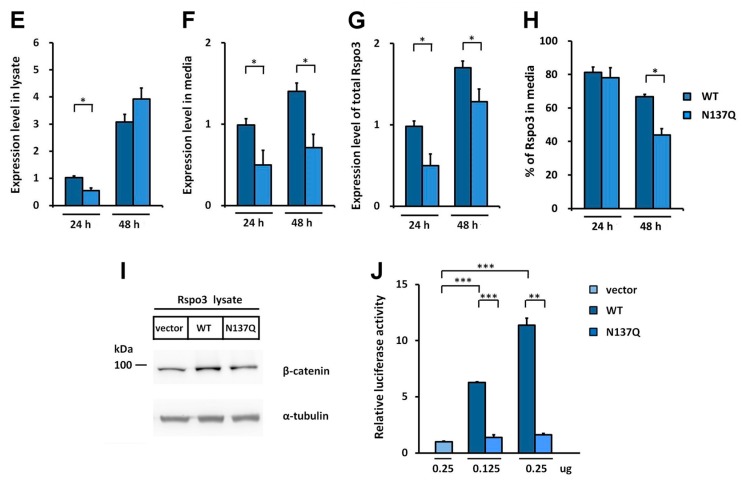
N-linked glycosylation of human Rspo3 is also required for its efficient secretion from cells. (**A**) Cell lysates and conditioned media from control vector or HA-tagged human Rspo3-transfected HEK293T cells were analyzed for the expression of HA-tagged human Rspo3 and endogenous soluble β-catenin by Western blot; (**B**) WT Rspo3 was transiently expressed in HEK293T cells in the presence of tunicamycin (TUN). The cell lysates and conditioned media were analyzed by Western blot; (**C**) The cell lysates and conditioned media of human Rspo3-transfected HEK293T cells were treated with (+) or without (−) PNGase F and subjected to Western blot analysis; (**D**) WT Rspo3 or N137Q mutant was transfected into HEK293T cells. After 24 or 48 h, the cell lysates and conditioned media were analyzed for HA-tagged Rspo3; (**E**–**H**) Quantification of relative expression levels for human WT Rspo3 *versus* N137Q mutants in cell lysates, media and total. Immunoblots were quantified densitometrically and relative expression level of Rspo3 in media or lysate was calculated. Percentage of Rspo3 in media was defined as the Rspo3 in the media as a percentage of the total Rspo3 (lysates plus media). Error bars represent the mean ± SD; (**I**) WT human Rspo3 or mutant N137Q plasmid DNA was transfected into HEK293T cells. After 48 h, the cell lysates were analyzed for soluble β-catenin; (**J**) SuperTopFlash plasmids were co-transfected with the indicated amounts of control vector, WT or N137Q Rspo3-expressing plasmids. Renilla luciferase was included as a transfection control. Error bars represent the mean ± SD. All experiments were performed at least two times, in triplicate. The Student’s *t*-test was used to evaluate statistical significance (* *p* < 0.05; ** *p* < 0.01; *** *p* < 0.001). α-tubulin was used as a loading control for the cell lysates in each blot.

Because N137 is also conserved among most Rspo3 members (Figure 2A,B), we next explored whether this site is also N-glycosylated in human Rspo3. When HA-tagged human Rspo3 was expressed in HEK293T cells, multiple immunoreactive bands ranging from 36 to 42 kDa were detected in the cell lysates and conditioned media (Figure 4A). All the immunoreactive bands are larger than the predicted molecular weight of 32 kDa, indicating possible post-translational modification. Secreted Rspo3 was active in enhancing Wnt/β-catenin activity as shown by the stabilisation of β-catenin in transfected cells (Figure 4A). Tunicamycin treatment of Rspo3-expressing cells led to reduced molecular masses in both the cell lysates and the conditioned media, suggesting that human Rspo3 is also an N-glycosylated protein *in vivo* (Figure 4B). Deglycosylation of the cell lysates or the conditioned media from transfected cells treated with PNGase F further confirmed these results. PNGase F digestion caused a similar molecular weight shift as observed with tunicamycin treatment, suggesting that human Rspo3 is also N-glycosylated in HEK293T cells (Figure 4C).

The conserved N137 sequon is one of four sequons found in human Rspo3 (Figure 2B). To explore whether the conserved N137 site was also N-glycosylated in Rspo3, an Rspo3 N137Q mutant was created and transfected it into HEK293T cells.

The N137Q mutant exhibited reduced molecular masses in both the cell lysates and the media compared with those in WT Rspo3 (Figure 4D). Similar to what was performed in Rspo1, the time course analysis also showed that mutation of N-glycosylation site at N137 significantly reduced the amount of proteins in media at 48 h post-transfection (% in media: WT, 67%; N137Q, 44%) (Figure 4E–H). Concomitant with the reduction in the secreted protein, the amount of soluble β-catenin was also reduced in the cell lysates of N137Q mutant cells compared with WT Rspo3 cells (Figure 4I). In addition, when co-transfected with a STF reporter plasmid, WT Rspo3 was more active than N137Q mutant (Figure 4J). Because of the intrinsic amplification property of the luciferase enzymatic reporter system, the result of reporter assay showed more pronounced difference than that of Western blot (Figure 4I,J). The above results corroborated the result that Rspo3 N137Q is present in lower amount in media than WT. Taken together, these results demonstrate that N-glycosylation at the conserved N137 site in human Rspo1 and Rspo3 is essential for their accumulation in media.

### 2.4. Introduction of the Rspo1 N-Glycosylation Site Enhances the Accumulation of Human Rspo2 in Media and Rescues the Accumulation Defect of Nonglycosylated Rspo2

**Figure 5:**
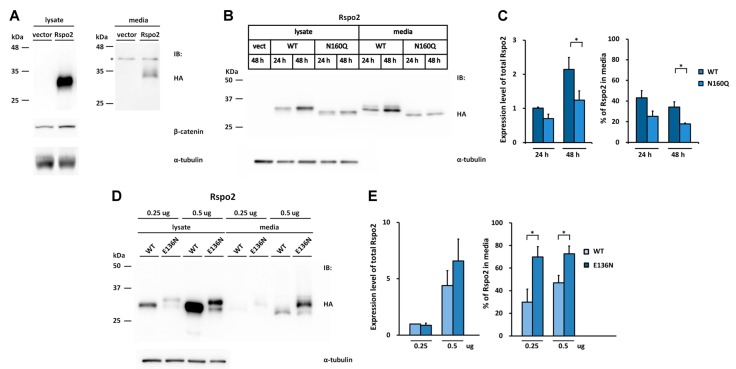

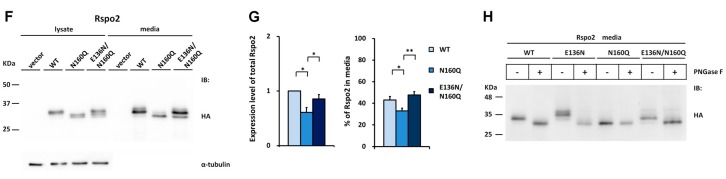
Effect of the engineered glycosylation site on glycosylation and secretion of human Rspo2. (**A**) Cell lysates or conditioned media from control vector or HA-tagged human Rspo2-transfected HEK293T cells were analyzed for the expression of HA-tagged human Rspo2 and the soluble β-catenin, * denotes non-specific bands; (**B**,**D**,**F**) WT Rspo2 or the indicated mutant was transfected into HEK293T cells. After 24 (**B**) or 48 h (**B**,**D**,**F**), the cell lysates and conditioned media were analyzed for HA-tagged Rspo2; (**C**,**E**,**G**) Quantification of total expression levels and percentage of Rspo2 in media for WT Rspo2 *versus* the indicated mutants are shown. Immunoblots were quantified densitometrically and relative expression level of Rspo2 in media or lysate was calculated. Error bars represent the mean ± SD. All experiments were performed in triplicate; (**H**) PNGase F digestion of WT, E136N, N160Q, and E136N/N160Q Rspo2 mutant. The Student’s *t*-test was used to evaluate statistical significance (* *p* < 0.05; ** *p* < 0.01). α-tubulin was used as a loading control for the cell lysates in each blot.

All the vertebrate Rspo2 members contain a sequon distinct from those found in Rspo1 and Rspo3. This sequon, N160, is located in the N-terminus of TSR1 domain (Figure 2A,B). When human HA-tagged Rspo2 was transfected into HEK293T cells, one single band with 32 kDa was detected in the cell lysates and conditioned media (Figure 5A). This molecular weight is larger than the predicted molecular weight of 29 kDa, indicating possible post-translational modification. Similar to those found in Rspo1 and Rspo3, secreted Rspo2 was active in potentiating canonical Wnt signaling, as evidenced from the accumulation of stabilized β-catenin in transfected cells (Figure 5A). Deglycosylation of the conditioned media from the Rspo2-transfected cells with PNGase F resulted in a reduction in molecular mass, suggesting that human Rspo2 is N-glycosylated in cells (Figure 5H). To determine the N-glycosylation site in Rspo2, a N160Q mutant was created by replacement of Asn160 with Gln. N160Q Rspo2 mutant exhibited a reduced molecular mass similar to that of PNGase F-treated WT Rspo2, suggesting that Rspo2 is N-glycosylated at N160 (Figure 5H). Resistance of PNGase F digestion of N160Q mutant indicated that N160 is the only N-glycosylation site in Rspo2 (Figure 5H). Time course analysis indicated that abrogation of N-glycosylation at N160 of Rspo2 resulted in a significant reduced level of Rspo2 in media at 48 h post-transfection when compared with that of WT Rspo2 (% in media: WT, 34%; N160Q, 18%) (Figure 5B,C).

We next explored whether N-glycosylation at the position homologous to N137 in Rspo1 and Rspo3 might have any effect on the accumulation of human Rspo2 in media. To this end, the Asn136 N-glycosylation site was introduced into the human Rspo2 by replacing Glu136 with Asn (E136N) to create the sequon Asn136-Glu137-Thr138.

When expressed in cells, the E136N Rspo2 mutant exhibited an increased molecular mass relative to the WT Rspo2. This molecular difference was abolished by PNGase F treatment of the E136N, confirming N-glycosylation at the N136 site of the mutant (Figure 5H). In addition, E136N Rspo2 mutant displayed a maximal two-fold increase of the amount present in media compared with that of WT (Figure 5D,E). To test whether N-glycosylation at this new sequon might improve the accumulation of nonglycosylated N160Q mutant in media, we further generated the N160Q/E136N mutant. Analysis of the lysates and conditioned media from the cells transfected with the WT, N160Q or E136N/N160Q Rspo2 mutant indicated that the N-glycosylation at N136 of Rspo2 fully rescued the accumulation defect of nonglycosylated N160Q Rspo2 mutant (Figure 5F,G). The presence of N-glycan in E136N/N160Q mutant was confirmed by PNGase F digestion (Figure 5H). Taken together, the result further supports the importance of N-glycan at the end of FR2 domain in the folding and media accumulation for Rspo protein family.

### 2.5. Introduction of the Rspo2 N-Glycosylation Site Rescues the Accumulation Defects of Nonglycosylated Rspo1 and Rspo3 Mutants

**Figure 6:**
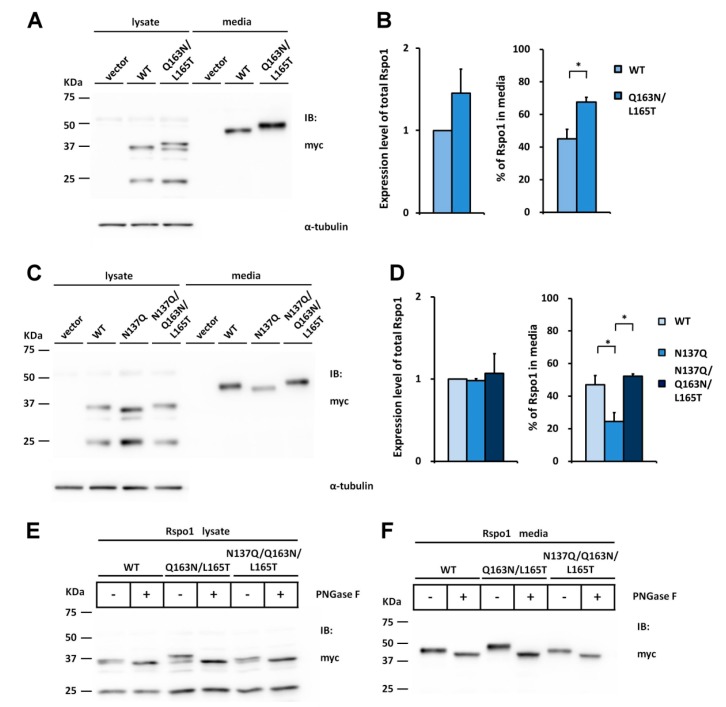
Introduction of the Rspo2 N-glycosylation site rescues the secretion defect of nonglycosylated human Rspo1 mutant. The Asn163-Gln164-Thr165 sequon (the position homologous to N160 in Rspo2) was constructed by mutation Q163N/L165T in the WT or nonglycosylated human N137Q Rspo1 mutant. (**A**,**C**) Cell lysates or conditioned media from control vector or myc-tagged human Rspo1-transfected HEK293T cells were analyzed for the expression of myc-tagged human Rspo1. α-tubulin was used as a loading control for the cell lysates; (**B**,**D**) Quantification of total expression levels and percentage of Rspo1 in media for human WT Rspo1 *versus* the indicated mutants are shown. Immunoblots were quantified densitometrically and percentage of Rspo1 in media was calculated as described previously. Error bars represent the mean ± SD. All experiments were performed in triplicate. The Student’s *t*-test was used to evaluate statistical significance (* *p* < 0.05); (**E**,**F**) Cell lysates and conditioned media from WT, Q163N/L165T or N137Q/Q163N/L165T mutant Rspo1-transfected cells were digested with PNGase F.

To determine whether N-glycosylation at N160 of Rspo2 is also important for folding and media accumulation in other Rspo members, we first introduced the N-glycosylation site of Rspo2 into the WT Rspo1 by replacing Gln163 and Leu165 with Asn and Thr, respectively (Q163N/L165T), resulting the sequon Asn163-Gln164-Thr165. Western blot analysis indicated that the introduced N-glycosylation site was fully glycosylated as evidenced from the slower mobility of the Q163N/L165T mutants relative to the WT Rspo1 (Figure 6E,F). The mobility difference was abolished by PNGase F treatment, confirming glycosylation of N163 (Figure 6E,F). Rspo1 with an engineered N-glycosylation site (Q163N/L165T) displayed an average 1.5-fold increase in the percentage of Rspo1 in media as compared with that of WT (Figure 6A,B). To test whether N-glycosylation at this new position might improve accumulation efficiency of nonglycosylated N137Q mutant, a N137Q/Q163N/L165T mutant was created and transfected into cells. Western blot analysis indicated that introduction of N-glycan at N163 on the nonglycosylated N137Q Rspo1 mutant fully restored its accumulation defect. The percentage in media was restored from 25% (N137Q) to 52% (N137Q/Q163N/ L165T), a similar level to that of WT (47%) (Figure 6C,D). PNGase F digestion of N137Q/Q163N/L165T mutant confirmed the occurrence of N-glycosylation (Figure 6E,F).

**Figure 7:**
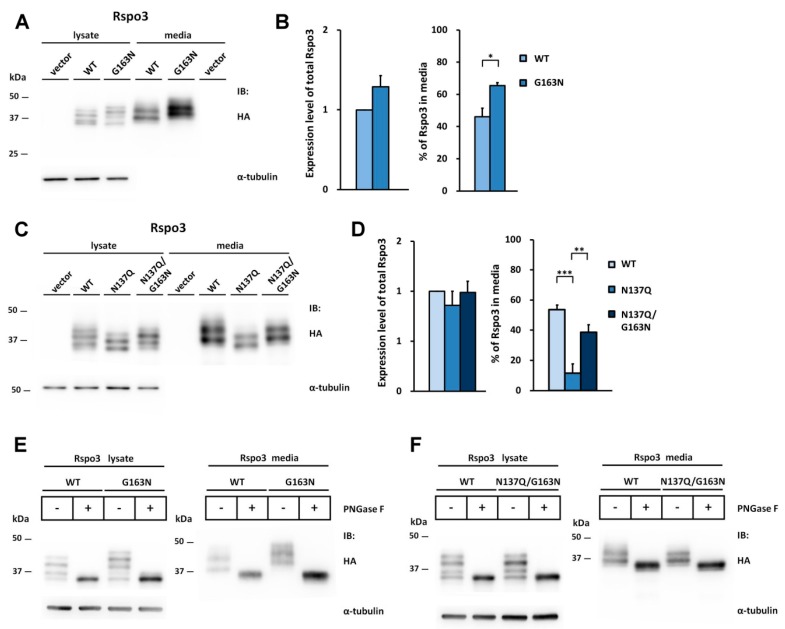
Introduction of the Rspo2 N-glycosylation site rescues the secretion defect of nonglycosylated human Rspo3 mutant. The Asn163-Lys164-Thr165 sequon (the position homologous to N160 in Rspo2) was constructed by mutation G163N in the WT or nonglycosylated human N137Q Rspo3 mutant. (**A**,**C**) Cell lysates or conditioned media from control vector or HA-tagged human Rspo3-transfected HEK293T cells were analyzed for the expression of HA-tagged human Rspo3. α-tubulin was used as a loading control for the cell lysates; (**B**,**D**) Quantification of total expression levels and percentage of Rspo3 in media for human WT Rspo3 *versus* the indicated mutants are shown. Immunoblots were quantified densitometrically and percentage of Rspo3 in media was calculated as described previously. Error bars represent the mean ± SD. All experiments were performed in triplicate. The Student’s *t*-test was used to evaluate statistical significance (* *p* < 0.05; ** *p* < 0.01; *** *p* < 0.001); (**E**,**F**) Cell lysates and conditioned media from WT, G163N or N137Q/G163N mutant Rspo3-transfected cells were digested with PNGase F.

To explore whether similar situation will occur in the case of Rspo3, we also introduced the N-glycosylation site to WT or Rspo3 mutant at the position homologous to N160 in Rspo2 by replacing Gly163 with Asn (G163N or N137Q/G163N) to create the sequon Asn163-Lys164-Thr165. As seen on the Western blot, an average 1.4-fold increase was found in the percentage of Q163N/L165T in media as compared with that of WT Rspo3 (Figure 7A,B). In addition, glycosylation on Asn163 partially rescued the accumulation defect of the nonglycosylated N137Q Rspo3 mutant (Figure 7C,D). PNGase F digestion confirmed that the higher molecular masses of the G163N mutants were caused by N-glycosylation and N137Q/G163N mutant had fully restored its glycosylation (Figure 7E,F).

Taken together, introduction of the N-glycosylation site at the position homologous to N160 in Rspo2 efficiently rescues the accumulation defect of nonglycosylated Rspo1 and Rspo3 mutant, suggesting that N-glycosylation at N137 and N160 both play an important role in efficient folding and media accumulation of Rspo protein family.

### 2.6. Effect of N-Glycans on the Stability of Intracellular Rspo1

**Figure 8:**
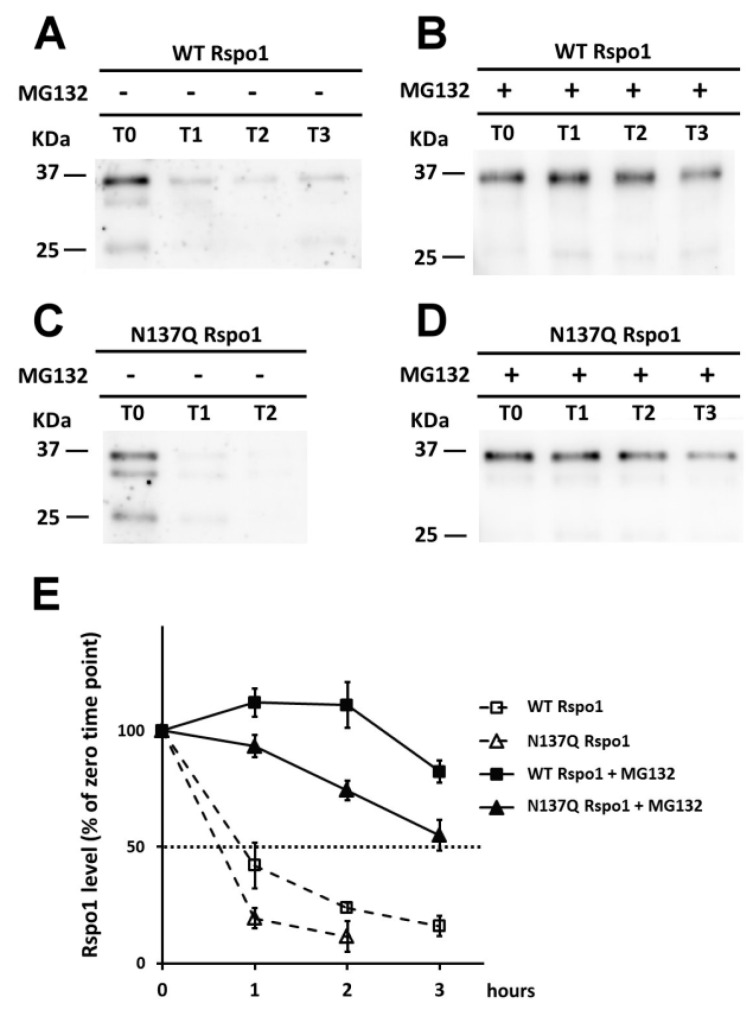
Effect of N-glycan on the stability of intracellular Rspo1. (**A**–**D**) HEK293T cells were transfected either with WT or N137Q Rspo1. After 24-h post-transfection, cells were labeled with azidohomoalanine and chased at the indicated time points in the presence or absence of MG132. Lysates were collected and conjugated with biotin via click chemistry reaction. Biotinylated proteins were then enriched with streptavidin-agarose and subjected to Western blot analysis using anti-Rspo1. After enhanced chemiluminescence (ECL) development, the same blot was stripped and reprobed with streptavidin-peroxidase for detection of total biotinylated proteins (Figures S1 and S2); (**E**) The densitometric values of Rspo1 were normalized to those of total biotinylated protein for comparison. The intensity of western blot for WT and N137Q Rspo1 at each time point was compared to the level of Rspo1 (100%) at the starting time (0 h). Error bars indicated the SD.

Misfolded protein and unfolded proteins, such as non-glycosylated mutants, in the ER are targeted for degradation through an ubiquitin-proteasome dependent mechanism known as ER-associated degradation (ERAD) [40]. To explore the possible fate of non-glycosylated N137Q Rspo1 mutant through ERAD pathway, we next performed pulse-chase labeling in Rspo1-transfected cell with azidohomoalanine (AHA), a biorthogonal noncanonical amino acid that acts as a methionine surrogate in newly synthesized protein [41]. Labeled cells were lysed and newly synthesized proteins bearing AHA were ligated to the biotin-tag. Newly synthesized biotinylated proteins were further enriched using streptavidin resin and subjected to Western blot analysis. The result showed that non-glycosylated N137Q mutant was unstable and degraded faster than WT Rspo1 (t1/2 for Rspo1: WT, 0.9 h; N137Q, 0.6 h) (Figure 8A,C,E). As anticipated, the degradation of WT Rspo1 and N137Q were both alleviated by addition of the proteasome inhibitor MG132 (Figure 8B,D), confirming that the proteasome is required for degradation of unglycosylated N137Q Rspo1. The results demonstrated that addition of N-glycan on N137 of Rspo1 affect its intracellular stability.

### 2.7. Effect of N-Glycan on the Stability of Secreted Rspos

**Figure 9:**
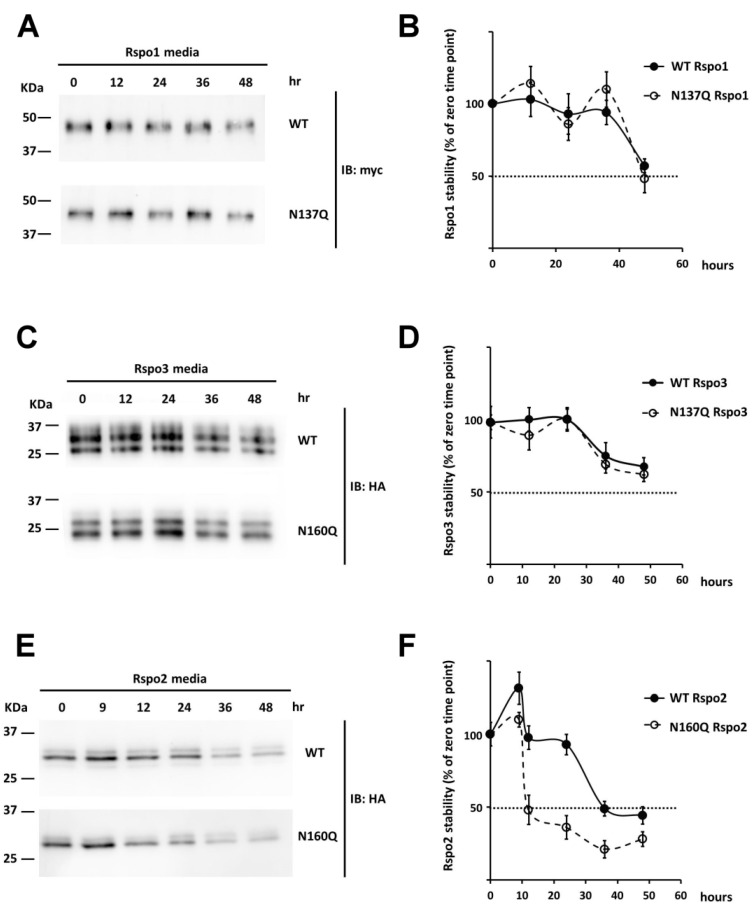
Effect of N-glycan on the stability of secreted Rspo1, 2 and 3. (**A**,**C**,**E**) Conditioned media containing WT Rspos and their variants were incubated at 37 °C for the indicated time points prior to Western blot analysis; (**B**,**D**,**F**) Quantification of the intensity for WT Rspos and their variants at each time point was compared to the level of Rspo (100%) at the starting time (0 h). Error bars indicated the SD.

In addition to affecting intracellular stability, addition of N-linked glycans to proteins can influence extracellular stability as well [42,43]. To determine the effect of N-glycans on the stability of secreted Rspos, serum-free conditioned media of Rspo-transfected cells were harvested and incubated at 37 °C. At the indicated time points, equal amount of each Rspo was denatured and subjected to Western analysis. Rspo1 and Rspo3 showed time-dependent degradation after 36 h and 24 h, respectively. The degradation of N137Q Rspo1 and N137Q Rspo3 was only slightly faster than their WTs (*t*_1/2_ for Rspo1: WT, >48 h; N137Q, ~48 h; *t*_1/2_ for Rspo3: WT and N137Q > 48 h) (Figure 9A–D). In contrast, unglycosylated Rspo2 mutants (N160Q Rspo2) showed more rapid degradation than WT (*t*_1/2_ for Rspo2: WT, 36 h; N160Q, 12 h) (Figure 9E,F). Overall, these data suggest that the N-glycan at N160 of Rspo2 affects the stability of secreted Rspo2, but N-glycans at N137 of secreted Rspo1 and Rspo3 have little effect on their stability.

### 2.8. Prevention of N-Glycosylation at N137 in Human Rspo1 and Rspo3 Did Not Affect Their Heparin-Binding Ability

**Figure 10:**
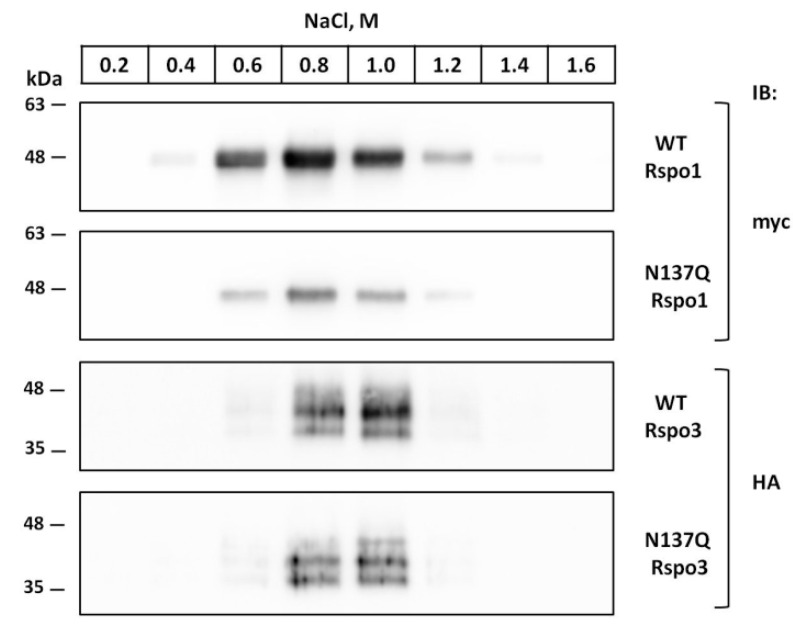
Effect of N-glycosylation on the heparin-binding ability of Rspo1 and Rspo3. Concentrated conditioned media from WT Rspo1, WT Rspo3 and their glycomutants-expressing cells were incubated with heparin-agarose, and the bound proteins were eluted with buffers containing increasing concentrations of NaCl. The presence of Rspo proteins in each eluate was detected by immunoblot analysis using anti-myc or anti-HA antibodies. Data shown here included a representative image of two independent experiments with similar results.

Previous research indicated that *E. coli*-derived Rspo CRD fragment, containing no other modification, could elicit similar potency of Wnt activity as mammalian cell-derived Rspos [44]. This result suggested that N-lined glycan is dispensable for the activity of Rspos. However, since the conservative N137 is near the TSR1 domain, which provides affinity towards HSPG [4,8], we were wondering whether N-glycosylation at N137 might had any contribution to heparin-binding ability. To examine the interactions between Rspos and heparin, conditioned media were absorbed to heparin-agarose and sequentially washed with buffers containing increasing amounts of NaCl, and Rspos was detected in the eluates by immunoblot. Consistent with previous findings [4], WT Rspo1 and WT Rspo3 bound to the heparin-agarose with high affinity and were eluted from the heparin agarose between 0.6 and 1.2 M NaCl (Figure 10). Similar elution profiles can be observed in their glycomutants (N137Q Rspo1 and N137Q Rspo3), suggesting that N-glycan at N137 in Rspo1 and Rspo3 did not contribute to their heparin-binding ability.

## 3. Discussion

R-spondins and their receptors, Znrf3/Rnf43 and type 2 Lgrs, play essential roles in Wnt signaling to regulate several biological processes, including stem cell biology [12,13,14] and bone density [45,46]. Therefore, Rspo1 is valuable in applications of regenerative medicine and holds therapeutic potential for treating diseases such as cancer and osteoporosis. Moreover, understanding the biogenesis of Rspo is crucial for the production of therapeutic recombinant proteins. In the present study, we found that human Rspo1 is N-glycosylated at N137. The secreted mature Rspo1 contains complex oligosaccharides as evidenced from the observation that Rspo1 in the conditioned media is Endo-H resistant. In addition, Rspo2 and Rspo3 are also N-glycosylated in cells. While Rspo1 and Rspo3 are both N-glycosylated at N137 residues, Rspo2 is at N-glycosylated at N160 site. Abrogation of N-glycosylation at these sites resulted in decreased level of Rspos accumulation in media. Rspo family belongs to a member of heparin binding proteins. Though N-glycosylation at N137 of Rspo1 and Rspo3 affects their level of media accumulation, it did not affect the binding ability to heparin*.*

Initially, we demonstrated that inhibiting the addition of N-glycans with tunicamycin substantially affected the secretion of Rspo1 and Rspo3. In addition, castanospermine treatment also compromised Rspo1 accumulation in media, suggesting that the interaction of Rspo1 with lectin chaperones, such as calnexin and calreticulin, was essential for the effective processing of Rspo1.

N-linked glycosylation plays a pivotal role during the maturation and secretion of many proteins [26], and the N-glycosylation site in a glycoprotein appears to be conserved [47]. A recent work comparing N-glycosylation sites among different species showed that the sequon motifs containing glycosylated Asns are more conserved than the motifs that have nonglycosylated Asns in the same proteins [48]. This finding suggests that glycosylation is very important in the evolution of species and variations that cause changes in the glycosylation pattern of a protein can be detrimental. While the numbers of sequons varied between vertebrate Rspo1 and Rspo3 members, they all contained the sequon with the conserved Asn137. Indeed, prevention of N-glycosylation at this conserved residue in either human Rspo1 or Rspo3 led to defective secretion. The result further highlights the importance of N-glycosylation in the Rspo protein family.

Despite its critical role, N137 is not conserved among Rspo2 members. In Rspo2, N-glycan is added at N160, a site near the N-terminus of TSR1 domain. We found that this modification is also required for the accumulation of Rspo2 in media. Introduction of the N-glycosylation site to Rspo2 mutant at the position homologous to N137 in Rspo1 or 3 rescued the accumulation defect of nonglycosylated Rspo2 mutant in media. This data indicated that the addition of N-glycan at N137 plays a role in the folding and media accumulation of Rspo protein family. Intriguingly, introduction of the N-glycosylation site to Rspo1 or 3 at the position homologous to N160 in Rspo2 also fully or partially restored the accumulation of Rspo1 or Rspo3 glycomutant in media, respectively. Recent studies indicated that glycosylation is essential to the folding process of newly synthesized protein through influencing the local secondary structure of protein and rigidifying the amino acid residues proximal to the glycosylation site [49]. Since these two sites are either close to (N137 in Rspo1 and 3) or within the TSR1 domain (N160 in Rspo2), it seems that N-glycan addition around this region play an essential role the folding and secretion for this protein family.

Addition of N-glycan on newly synthesized proteins also contributes to quality control surveillance in the ER. Unfolded proteins and misfolded proteins, such as unglycosylated mutant proteins, will be retained in the ER and targeted to degradation by ERAD pathway [40]. Pulse-chase analysis revealed that non-glycosylated mutant, N137Q Rspo1, showed a more rapid degradation rate than WT Rspo1 in cells. Treatment of the proteasome inhibitor MG132 markedly slowed the degradation rate of N137Q Rspo1 in cell lysate. This result indicated that human Rspo1 is subjected to ER quality control in N-glycan-dependent manner. While N-glycan plays a role in the intracellular stability of Rspo1, it had little effect on secreted Rspo1. The stability analysis of secreted Rspo1, 2 and 3 showed that N-glycans at N137 of secreted Rspo1 and Rspo3 have little effect on their stability, whereas N-glycan at N160 of Rspo2 has greater effect on the stability of secreted Rspo2. Why does N-glycan at secreted Rspo2 play a greater role in stability than that in Rspo1 and 3? In our preliminary observation, secreted Rspo1 and Rspo3 both contain mucin-type *O*-glycosylation, which is not present in secreted Rspo2 [50]. Mucin-type *O*-glycosylation has been known to play a role in protein stability [51]. Whether this type of modification might contribute to the stability of secreted Rspo1 and 3 is left for further study.

All Rspo family members contain a single TSR1 domain that binds to heparin or HSPGs [4]. Recent studies have indicated that two forms of domain-specific glycosylation are found in the TSR1 domain, namely, *C*-mannosylation and *O*-fucosylation [52]. Further study is ongoing to elucidate the types of modification other than N-glycosylation and their role(s) in Rspo biogenesis.

**Figure 11:**
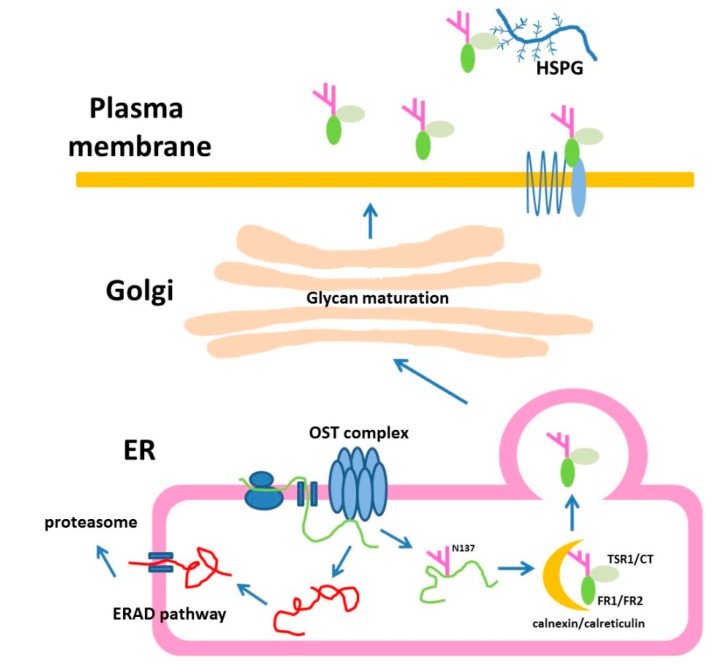
Model for N-glycosylation of Rspo1 in cells. Rspo1 is N-glycosylated at N137, a site near C-terminus of FR2, through oligosaccharyltransferase (OST) complex. Alternatively, this N-glycosylation site can be replaced with the position homologous to N160 in Rspo2 (Q163N/L165T Rspo1), a site near the N-terminus of TSR1. N-glycan of Rspo1 is further trimmed through glucose removal by α‑glucosidase. The trimmed Rspo1 then becomes a ligand for ER lectins, calreticulin and calnexin. After the assisted folding with lectins, the Rspo1 becomes properly folded in the ER. Correctly folded N-glycosylated Rspo1 are packaged for transport to the Golgi. After translocation to the Golgi*,* the oligosaccharide chains are further processed. Unglycosylated mutants N137Q Rspo1 is targeted for degradation through an ubiquitin-proteasome dependent mechanism known as ERAD. The mature secreted Rspo1 is composed of complex type of oligosaccharides. Secreted Rspo1 can bind to LGR4/5/6 and ZNRF3/Rnf43 receptors complex to potentiate Wnt signal through CRD domain (FR1/FR2). Alternatively, their TSR1 and CT domains can provide affinity towards heparan sulfate proteoglycan *(*HSPG*).*

In summary, our results provide evidences for the critical role of N-glycans in determining the secretion of Rspo family, especially on Rspo1. Based on our results, the following model is proposed for N-glycosylation of Rspo1 protein during its biogenesis (Figure 11). In summary, these data contribute to an increased understanding of the biogenesis of Rspo family members, especially on the role of N-glycosylation at two particular sites in the folding, stability and secretion.

## 4. Experimental Section

### 4.1. Plasmid Construction and Site-Directed Mutagenesis

The vector expressing human Rspo1 with a myc-tag (pCMV6-hRspo1-myc-ddk) was purchased from Origene (Rockville, MD, USA). The cDNA encoding human Rspo2 and Rspo3 was purchased from Sino Biological Inc. (Beijing, China) and Openbiosystem (Huntsville, AL, USA), respectively. The coding sequences for human Rspo2 or Rspo3 were subcloned into pCS2 by PCR, resulting in the expression plasmid for Rspo2 or Rspo3 with a C-terminally HA-tag (pCS2-hRspo2-HA or pCS2-hRspo3-HA). The primers used for subcloning are listed in Table S1. The firefly luciferase reporter plasmid SuperTopflash was obtained from Addgene (Cambridge, MA, USA), and the control renilla luciferase plasmid phRG-TK was obtained from Promega (Madison, WI, USA). To generate individual mutants, the QuickChange Lightning site-directed mutagenesis kit (Stratagene Inc., La Jolla, CA, USA) was used using the primer sets listed in Table S1.

### 4.2. Cell Culture and Transfection

HEK 293T cells were cultured in 12-well tissue culture plates (5 × 10^4^ cells per well) in Dulbecco’s modified Eagle’s medium (DMEM) (Gibco Life Technologies, Grand Island, NY, USA) supplemented with 10% foetal bovine serum (Hyclone Inc., Logan, UT, USA), 4 mM glutamine and 1% penicillin/streptomycin at 37 °C in a humidified atmosphere containing 5% CO_2_. Cells were transiently transfected using the T-pro NTR-II transfection reagent according to manufacturer’s protocol (T-pro Biotech., New Taipei County, Taiwan). Before transfection, the culture medium was replaced with Gibco Opti-MEM reduced serum medium (Gibco Life Technologies) (containing 2% foetal calf serum). After 24 or 48 h post-transfection, conditioned media and cells were collected for further biochemical analyses.

### 4.3. Cell Fractionation and Western Blot

The cell lysates were prepared by the addition of lysis buffer (50 mM Tris-HCl, pH 7.4, 150 mM NaCl, 0.5% sodium deoxycholate, 0.1% SDS, 2 mM EDTA, 50 mM NaF) containing a protease inhibitor cocktail (Complete™, Roche Inc., Penzberg, Upper Bavaria, Germany) and were incubated at 4 °C for 15 min. After vortexing, the cell homogenates were subjected to centrifugation at 10,000× *g* for 20 min at 4 °C, and the supernatant was collected. The protein concentrations of the samples were determined with the Bradford dye binding assay (Bio-Rad Inc., Hercules, CA, USA) using bovine serum albumin (Sigma, St. Louis, MO, USA) for the standards. Soluble cell lysates or conditioned media were denatured by the addition of an appropriate amount of 4x SDS loading buffer (240 mM Tris-HCl, pH 6.8, 8% SDS, 5% β-mercaptoethanol, 40% Glycerol, 0.04% Bromophenol Blue) and heated in boiling water for 5 min. Denatured proteins were loaded onto 10% or 12.5% SDS-PAGE gels (Bio-Rad Mini-Protean Electrophoresis system, 10.1 cm × 7.3 cm with 1.5 mm spacer) and separated. After separation, the proteins on the gel were subsequently transferred to polyvinylidene difluoride membranes (PVDF, Pall Corporation, Port Washington, NY, USA) using a Mini Trans-blot module (Bio-Rad). The membranes were blocked for 1 h at room temperature in blocking buffer (BlockPro^TM^ blocking buffer, Visual Protein Biotechnology, Taipei, Taiwan) and then incubated overnight with the appropriate dilution of primary antibody in blocking buffer. After six washes in TBST for 5 min each, the blots were incubated with secondary antibody diluted in blocking buffer for 1 h at room temperature, washed six times in TBST for 5 min each, and developed using the T-pro LumiLong Chemiluminescence Detection kit (T-pro Biotech) according to the manufacturer’s protocol. The chemiluminescence signal was detected using ImageQuant™ LAS 4000 (GE Health, Little Chalfont, UK), and the densitometric quantification of the exposed images was performed with MultiGauge software (Version 2.2, Fujifilm, Minato, Tokyo, Japan). To quantitate the relative amount of Rspo(s) expressed in the cell and the conditioned media, the lysates and conditioned media were run on the same gel using the following loading amounts. For Rspo1, 4 μg of cell lysate and 15 μL of media were loaded. For Rspo2, 1 μg of cell lysate and 11 μL of media were loaded. For Rspo3, 2 μg of cell lysate and 15 μL were loaded. The above loading condition was chose so that their relative signal can be detected on the same blot and densitometrically quantitated. Since the total amount of protein lysate and the total volume of conditioned media in each transfected cell were known, then the relative levels of Rspo1 in cell lysate, media and the total (cell lysate plus media) can be obtained. The antibodies used in this study included mouse monoclonal anti-myc antibody (Abgent, Shanghai, China, 1:2000, for Rspo1,), mouse monoclonal anti-HA antibody (Covance, Princeton, NJ, USA, 1:3000, for Rspo2), rabbit polyclonal anti-HA antibody (Santa Cruz Biotechnology, Santa Cruz, CA, USA, 1:1500, for Rspo3), mouse monoclonal anti-β-catenin antibody (BD Biosciences, San Jose, CA, USA, 1:3000), mouse monoclonal anti-α-tubulin antibody (Novus, Littleton, CO, USA, DM1A, 1:4000), goat anti-mouse antibody conjugated with horseradish peroxidase (Jackson ImmunoResearch, Baltimore, MD, USA, 1:3000), and goat anti-rabbit antibody conjugated with horseradish peroxidase (Jackson ImmunoResearch, 1:3000).

### 4.4. Chemical Treatment and Deglycosylation

For chemical treatments, tunicamycin (Enzo, Farmingdale, NY, USA) or castanospermine (Enzo) was added to the media at a final concentration of 2.5 or 100 μg/mL, respectively, at 2 h post-transfection. Deglycosylation of the samples with PNGase F or Endo H (New England Biolabs, Ipswich, MA, USA) was performed according to manufacturer’s instructions. Digestion with the enzymes was carried out at 37 °C overnight. Samples were then denatured in SDS loading buffer and subjected to Western blot analysis.

### 4.5. SuperTopFlash Reporter Assay

HEK 293T cells were cultured in 12-well tissue culture plates as described above. SuperTopFlash plasmids (40 ng per well) were co-transfected with the indicated amounts of either the control vector or the indicated plasmids. Renilla luciferase, driven by the thymidine kinase gene promoter, was also included as a transfection control (20 ng per well). After transfection for 48 h, cell lysates were prepared by the addition of passive lysis buffer provided in the Dual-Luciferase Reporter Assay System kit (Promega) and clarified by centrifugation at 10,000× *g* for 15 min at 4 °C. The supernatant was then analyzed for both firefly luciferase activity and renilla luciferase activity according to the manufacturer’s protocol.

### 4.6. Pulse-Chase Experiments

Pulse-chase experiments were performed after HEK293T cells transfected with either WT- or N137Q mutant-expressing plasmid for 24 h. Cells were starved by replacing culture media with methionine-free DMEM (Invitrogen, Waltham, MA, USA) for 30 min, incubated with DMEM containing 50 μM azidohomoalaline (AHA) for 40 min, washed with phosphate buffer saline (PBS), and cultured for indicated times with Opti-MEM (Invitrogen, Inc*.*) in the presence or absence of MG132 (Enzo, 10 μM). Cell lysates were collected at indicated time after labeling. Labeled proteins were biotinylated with biotin-alkyne via click chemistry reaction using Click-iT^®^ Cell Reaction Buffer Kit (Thermo Fisher Scientific, Waltham, MA, USA). Samples were dialyzed overnight in PBS to remove excess biotin-alkyne reagent. Biotinylated proteins were enriched using streptavidin-agarose resin (NeutriAvidin, Pierce, Rockford, IL, USA). These newly synthesized proteins (biotinylated proteins) were then separated by SDS-PAGE and subjected to Western blot analysis using anti-Rspo1 (rabbit polyclonal antibody, ProteinTech, Chicago, IL, USA, 1:500). Before antibody incubation, the blot was blocked using BlockPro^TM^ protein-free blocking buffer (Visual Protein Biotechnology). After enhanced chemiluminescence (ECL) development, the same blot was stripped and reprobed with streptavidin-peroxidase (Pierce, 1:1500) for detection of total biotinylated proteins.

### 4.7. Stability of Secreted Rspos

Serum-free conditioned media harvested from Rspo-transfected cells were incubated at 37 °C and collected at the indicated time points. Equal amounts of each Rspo were denatured and subjected to Western blot analysis. The resultant blot was analyzed by using quantitative densitometry, the intensity of WT and its glycomutant at each time point was compared to the level of Rspos (100%) at the starting time (0 h). From the densitometry analysis of Western blots, we estimated the half-life of WT Rspos and their variants in solution.

### 4.8. Heparin Binding Assay

To perform the assay, vector expressing either WT Rspo1, WT Rspo3 or their glycomutants was transfected into HEK293T cells and cultured using serum-free Opti-MEM (Life Technologies, Carlsbad, NM, USA). After 72 h, conditioned media from were concentrated using Microsep advance centrifugal device (Pall, Michigan, ND, USA) and absorbed to heparin agarose (Sigma) at 4 °C with rocking overnight. The agarose was pelleted by centrifugation and washed by sequential resuspensions in 50 mM Tris (pH 8.0) with the indicated amount of NaCl. Rspo present in each eluate was detected using immunoblot by anti-myc (for Rspo1) or anti-HA antibody (for Rspo3).

## Supplementary Materials

Supplementary materials can be found at http://www.mdpi.com/1422-0067/17/6/937/s1.
