# Supplementary material for: N-Glycosylation of Human R-Spondin 1 Is Required for Efficient Secretion and Stability but Not for Its Heparin Binding Ability
Source: Int J Mol Sci. 2016 Jun 14;17(6):937. doi: 10.3390/ijms17060937 (PMC4926470; doi:10.3390/ijms17060937)
Supplement: Supplementary file 1 [file ijms-17-00937-s001.pdf]

Supplementary Materials: N-Glycosylation of Human R-Spondin 1 Is Required for Efficient Secretion and Stability but Not for Its Heparin Binding Ability

Chiung-Fang Chang, Li-Sung Hsu, Chieh-Yu Weng, Chih-Kai Chen, Shu-Ying Wang, Yi-Hwa Chou, Yan-Yu Liu, Zi-Xiu Yuan, Wen-Ying Huang, Ho Lin, Yau-Hung Chen and Jen-Ning Tsai

Table S1. PCR Primers used for site-directed mutagenesis of human Rspo1, Rspo2 and Rspo3.

Primer Name	Primer Sequence
Rspo1(N137Q)-sense	5′-GCTCCTCAGCTGCCCAAGGCACCATGGAGTG-3′
Rspo1(N137Q)-antisense	5′-CACTCCATGGTGCCTTGGGCAGCTGAGGAGC-3′
Rspo1(Q163N)-sense	5′-GGAAACCACAGAGCTGATTCTTCTTGGAGCAGGGC-3′
Rspo1(Q163N)-antisense	5′-GCCCTGCTCCAAGAAGAATCAGCTCTGTGGTTTCC-3′
Rspo1(L165T)-sense	5′-CCCTCCGGAAACCACAGGTCTGATTCTTCTTGGAGC-3′
Rspo1(L165T)-antisense	5′-GCTCCAAGAAGAATCAGACCTGTGGTTTCCGGAGGG-3′7Y8
Rspo2(E136N)-sense	5′-CCACACATTCCATGGTTTCGTTTAATGGTGCAAAACCATCTGG-3′
Rspo2(E136N)-antisense	5′-CCAGATGGTTTTGCACCATTAAACGAAACCATGGAATGTGTGG-3′
Rspo2(N160Q)-sense	5′-ATCCACATGTGCGTTGATTTCTGCTACAAGTTCCCCATTCG-3′
Rspo2(N160Q)-antisense	5′-CGAATGGGGAACTTGTAGCAGAAATCAACGCACATGTGGAT-3′
Rspo3(N137Q)-sense	5′-AGAAGGGTTGGAAGCCAACCAACATACTATGGAGTGTGTCA-3′
Rspo3(N137Q)-antisense	5′-TGACACACTCCATAGTATGTTGGTTGGCTTCCAACCCTTCT-3′
Rspo3(G163N)-sense	5′-CCTCTTTTGAAGCCACATGTTTTGTTCTTCTTCGTGCATGGACTCCAAG-3′
Rspo3(G163N)-antisense	5′-CTTGGAGTCCATGCACGAAGAAGAACAAAACATGTGGCTTCAAAAGAGG-3′

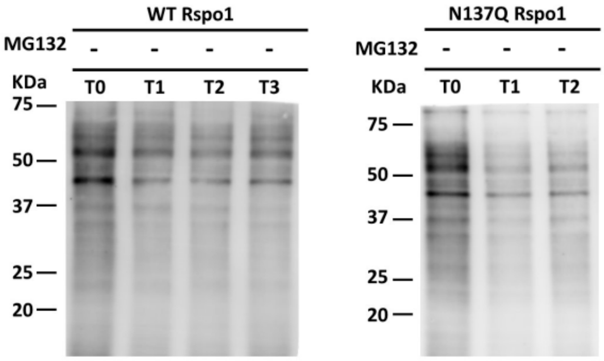

Figure S1. Detection of total biotinylated proteins (newly synthesized proteins) in the pulse-chase labeling of Rspo1-trasnfected cells in the absence of MG132.

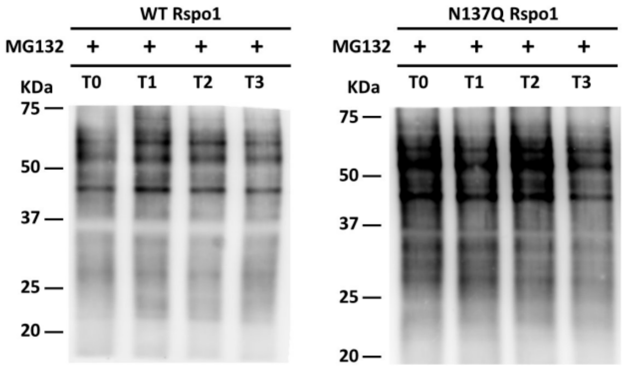

Figure S2. Detection of total biotinylated proteins (newly synthesized proteins) in the pulse-chase labeling of Rspo1-trasnfected cells in the presence of MG132.
